# Air Pollution and Migration Intention: Evidence from the Unified National Graduate Entrance Examination

**DOI:** 10.3390/ijerph19148813

**Published:** 2022-07-20

**Authors:** Chao Xu, Xiulei Wang

**Affiliations:** 1 School of Public Finance & Taxation, Shandong University of Finance and Economics, Jinan 250002, China; xuchaode@126.com; 2 School of Economics & Trade, Hunan University, Changsha 410006, China

**Keywords:** air pollution, migration intention, high human capital, instrumental variable

## Abstract

Using a unique dataset of applicants for the Unified National Graduate Entrance Examination (UNGEE) of 76 double first-class universities in China, this paper evaluates the causal impact of air pollution on the migration intentions of highly educated talents by exploiting an instrumental variable approach based on annually average wind speed. We find that a 1 ug/m^3^ increase in the annually average PM_2.5_ concentration in destination cities decreases the number of applicants for the UNGEE of elite universities by about 250, but better university quality and more abundant educational resources can weaken the effect partially. A heterogeneity analysis indicates that the university-city choices of applicants are shifting from north to south. Our findings suggest that air pollution may lead to the loss of high human capital.

## 1. Introduction

Air pollution has become increasingly deteriorated and attracted widespread attention worldwide, and is a major health hazard. According to the World Bank Report, air pollution is responsible for millions of deaths and creates economic loss which amounts to trillions of dollars in Asia and Sub-Saharan Africa (see http://www.yicai.com/news/5097668.html (accessed on 9 September 2016)). Mounting research has investigated the causal health effects of air pollution in both developed countries [1,2] and developing countries [3,4]. These studies find that acute exposure to air pollution can cause damage to physical and mental health, such as increasing mortality [5], increasing overweight and obesity rates [6], reducing life expectancy [7], and reducing hedonic happiness [8]. A growing body of literature also finds that people may adopt avoidance strategies, and even re-locate and migrate to reduce air pollution exposure in highly polluted areas [9,10,11]. Due to higher incomes and greater knowledge of the harmful effects of air pollution, elites are more sensitive to air pollution and have a higher willingness to pay for avoiding pollution [12,13]. However, little is known about the influence of air pollution on migration intentions of highly educated talents in developing countries.

Data on the applicants for the UNGEE of double first-class universities in China provide an opportunity to study the effects of air pollution on the migration intentions of highly educated talents. First, the UNGEE, or kaoyan, is a selective examination organized by the education authorities and admissions agencies for the selection of graduate students. The UNGEE is becoming more and more difficult as the number of applicants increases year by year. (In the past five years, the number of applicants for the UNGEE has increased rapidly. In 2017, for the first time, the number of applicants exceeded 2 million. In 2021, the number of applicants reached 3.77 million with an increase of 360,000 from 3.41 million in 2020, which has reached a record high. The number of applicants was 4.57 million in 2022. See http://www.moe.gov.cn/jyb_xwfb/s5147/202012/t20201228_507808.html (accessed on 26 December 2020)). The requirements and difficulties of the UNGEE of double first-class universities, China’s elite universities, are much greater than those of ordinary universities. Most applicants of double first-class universities are about to obtain or have already obtained bachelor’s degrees, which allows us to focus on highly educated talents. Second, the dataset on applicants only includes the number of applicants for full-time master’s degree students. If applicants are admitted as full-time postgraduate students, they must study for master’s degrees in university for the next two to three years. Therefore, the university-city choices represent their intentions to move over the next few years. In addition, compared with college-city choices after the National College Entrance Examination (gaokao), applicants are more likely to work and live in cities in which they apply for their master’s degrees in the future. Using a unique manually collected dataset on the applicants for UNGEE of 76 double first-class universities in China, this paper estimates the causal effects of air pollution on the migration intentions of highly educated talents. We exploit an instrument variable (IV) for air pollution to correct the estimated bias of the ordinary least square estimation (OLS) from omitted variables and measurement errors. The IV estimation results show that a 1 ug/m^3^ increase in PM_2.5_ concentration in destination cities significantly decreases the number of applicants by 250. This effect is equivalent to 2.2%, given the average 11,547 applicants. The magnitude of the result is consistent with the research on the job location decisions [14]. The reduction in the number of applicants in social science due to air pollution is larger than that in natural science. The results are consistent across different specifications and alternative samples. Moreover, heterogeneous effects show that air pollution decreases more applicants for the UNGEE of first-class discipline universities, which reveals that better university quality can remedy the negative effects partially. Furthermore, we also find that the university-city choices of applicants are shifting from north to south.

This paper makes three contributions to the existing literature. First, to the best of our knowledge, it is among the first to investigate the causal impacts of destination cities’ air pollution on migration intentions in highly polluted developing countries, using unique university-level applicant data. A large literature in environmental economics explores the negative effects of original and destination cities’ air pollution on job location decisions [12,14], and net-outmigration rates in China [11]. However, little is known about the relationship between air pollution and migration intentions. This paper is closest to that of Qin and Zhu (2018) and Jia and Chen (2021). They find that increases in original cities’ air quality index (AQI) increase the settlement intentions of floating migrants and emigration interests in China. We study the same linkage between air pollution and migration intentions, but we focus on the effects of different pollutants (PM_2.5_ versus AQI) on migration intentions of different groups (highly educated talents versus potential international migrants and floating migrants). Using an online search index on “emigration” to construct a daily measurement of city-level people’s emigration interests, Qin and Zhu (2018) examine the effects of air pollution on emigration interests with the Poisson regression model. Jia and Chen (2021) study the effects of air pollution on migrants’ settlement intentions with an instrumental variable approach using the ventilation coefficient. Based on survey questions from the 2017 National Migrant Population Dynamic Monitoring Survey, a dummy variable is constructed to measure the settlement intentions of floating migrants by Jia and Chen (2021). However, we exploit an instrumental variable based on wind speed and use the number of applicants for UNGEE of double first-class universities to measure short-term migration intentions of highly educated talents.

Second, this paper also adds to the literature on the determinants of migration decisions. A large body of studies have focused on economic and environmental factors such as income [15], wage [16], labor market demand [17] and housing prices [18]. There is some evidence of the causal relationship between air pollution and migration choices and the intentions of floating migrants [11] and the general population [19]. Few research studies have focused solely on highly educated groups. This study confirms previous findings that air pollution is associated with brain drain, not only from the perspective of job location choices of graduate students, but also from the perspective of location choices of studying for master’s degrees. Relative to previous literature on the job location choices of Tsinghua graduates and general college graduates [12,14], we focus on the applicants for UNGEE of 76 double first-class universities. Our sample includes more elite universities than Zheng et al. (2019) and is more concentrated on high quality universities than Lai et al. (2021).

Third, although many studies find that air pollution has negative effects on economic and social welfare from the perspective of productivity [20], human capital [21], crime [22], and physical and mental health [23,24], evidence of the causal relationship between air pollution exposure and talent loss is insufficient because of data availability. Highly educated talents are the core competitiveness of development in the future. The economic and social costs of the loss of talents are becoming increasingly apparent. Policy makers should focus on environmental amenities to attract talents, who are more sensitive to air pollution and climate change.

The rest of this paper is structured as follows. The Section 2 presents data and empirical strategy. Section 3 introduces the baseline results and provides robustness checks. Section 4 discusses the heterogeneous effects of air pollution on the quantity of applicants across the quality, type, and region of universities. Section 5 compares the results with the existing literature. Section 6 concludes.

## 2. Methodology

### 2.1. Data and Variables Analysis

**Figure 1:**
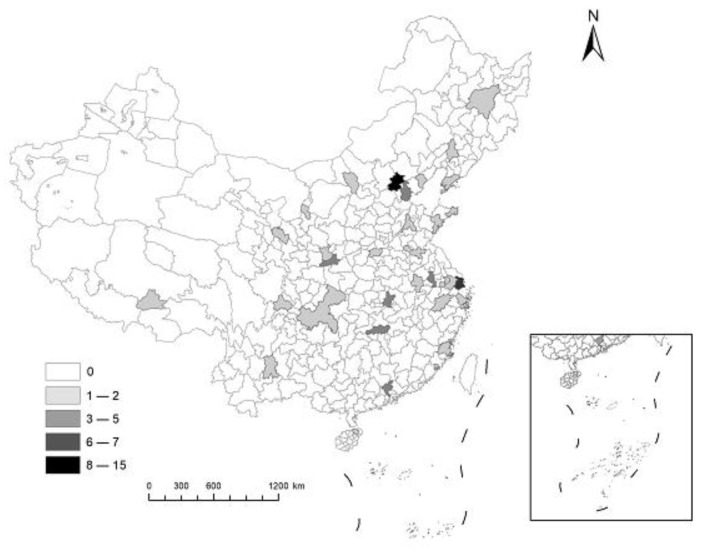
Regional distribution and quantity of sample universities in China. Note: This figure presents the regional distribution and quantity of all sample universities in China, and the different degrees of gray represent the different amounts of universities in each city.

It is plausible for us to define the applicants who apply for 76 double first-class universities of UNGEE as the elites. According to the requirements of the China Graduate Admission Information Network, the applicants for UNGEE must be the people who are about to obtain or have already obtained bachelor’s degrees, or have graduated from vocational college for over 2 years (see https://yz.chsi.com.cn/kyzx/jybzc/202109/20210903/2105941509.html (accessed on 3 September 2021)). However, for the applicants who graduated from vocational college, the aimed universities that they choose require them to have academic achievements in the journals of the Chinese Science Citation Database or Chinese Social Sciences Citation Index in the past 3 years, additionally. Therefore, most of the applicants who apply for UNGEE successfully are those who are about to or have obtained bachelor’s degrees. According to the data that was released by the National Bureau of Statistics of China, until 2019, the number of undergraduates in China accounted for only about 3.8% of the total population (see http://www.stats.gov.cn/ (accessed on 2 December 2020)). No matter the education degree or the population proportion, applicants who apply for UNGEE can be treated as elites in China. Under this premise, based on 137 double first-class universities that are listed in China’s fourth round of subject evaluation (see http://www.cdgdc.edu.cn/xwyyjsjyxx/xkpgjg/ (accessed on 28 December 2017)), and further considering whether the schools’ official websites have published the application data of past years for UNGEE, 76 universities are finally determined as the research sample (The specific list of 76 universities is shown in Table A1 in the Appendix A. Some universities have different campuses in different cities; in this paper, the campuses of the same universities in different cities are regarded as different universities). We manually crawl the data that include the quantity of applicants for 2010–2021, the number of students to be enrolled in the admission brochure for 2010–2021, and the enrollment ratio for 2009–2020. The amount of universities that we choose holds for 55.5% of all double first-class universities, and the geographical distributions of them cover 34 prefecture-level cities of 25 provinces in China. Figure 1 plots the specific regional distribution and number of double first-class university for every city. From the layout, the universities are locating in Shihezi of Xinjiang in the west, Ningbo of Zhejiang in the east, Harbin of Heilongjiang in the north, and Haikou of Hainan in the south, which have a generally representative in area. Owing to the fact that the data of some universities are missing in some years, the data that we use are an unbalanced panel data and the total amount is 585. Due to the need to examine the differences between applicants of different disciplines, and according to the “Catalogue of Disciplines and Majors for Granting Doctoral” and “Master’s Degrees and Cultivating Postgraduates” that are issued by the Ministry of Education of the People’s Republic of China (MOE of PRC) (see http://www.moe.gov.cn/srcsite/A08/moe_1034/s3882/201209/t20120918_143152.html (accessed on 14 September 2012)), we further divide the applicants into social science and natural science and then re-count the quantity of the different discipline applicants (social science includes: philosophy, economics, law, education, literature, history, military science, and management; natural science includes: science, engineering, agriculture, and medicine).

To determine the variables, we select the number of applicants for UNGEE in each university as the dependent variable (Apply) and choose the average urban PM_2.5_ concentration as the independent variable (PM_2.5_). As for the control variables, we mainly consider the following three aspects. The first is the factors of the university: when applicants choose a university in applying for UNGEE, they refer to the expectedly admitted student number that is published in the admission brochures of the aimed university firstly. The more the quantity of students to be recruited, the higher the probability of admission, and the more intense the intentions of students to apply are. Therefore, we add the number of students to be enrolled into the model as the control variable in school (Enrollment). The second is the characteristics of the city: the applicants also take urban features of the city where the intended university is located into consideration. A better urban economic development, richer recreational life, and lower living cost will be more attractive to applicants. Hence, we add per capita GDP (Rj_gdp), third industry proportion (Industry 3_rate), and consumer price index (CPI) into the equation. In addition, applicants will also consider comfort, convenience of urban life, and educational resource. So, the density of the population (Popu), the number of buses and trams (Transpor), and per capita educational expenditure (Edu_cost) are also added into the model. Last is the climate conditions of the city: different growth backgrounds make applicants have different climatic adaptability. If the applicants are admitted by the intended university, they will study and live in the city where the intended university is located as “quasi-residents” for 2 or 3 years, so the applicants will also consider the climatic features of the city when apply for UNGEE. We put annually average rainfall (Avgrain) and annually average temperature (Avgtemp) into the model. The application for UNGEE of the corresponding year was completed in October of the previous year, so the explanatory variables, except the expectedly admitted student number, are introduced into the model with a lag of one term.

The data of the universities are from official websites of 76 double first-class universities. The other data such as per capita GDP, consumer price index (CPI), third industry ratio, and so on are from the 2009–2020 “China Urban Statistical Yearbook”, and the urban PM_2.5_ concentration data are from the 2009–2020 PM_2.5_ density map released by Columbia University; the annually average temperature and annually average rainfall are from the China Meteorological Data Sharing Service System.

### 2.2. Descriptive Statistics

We use the “summary” command of Stata to perform the statistical analysis with the main variables that are used in this paper. The statistics include observation, mean, minimum, maximum, and standard deviation. Table 1 shows the summary statistics for the main variables used in the analysis. On average, the quantity of applicants for UNGEE in each university is about 11,547, and there is a big difference between various universities. Among them, there are relatively more applicants who major in social science, accounting for about 57.6%, and relatively fewer applicants for natural science, holding for around only 42.4%. The mean of urban PM_2.5_ concentration is approximate 49.79 ug/m^3^, and there are little differences between different cities.

**Figure 2:**
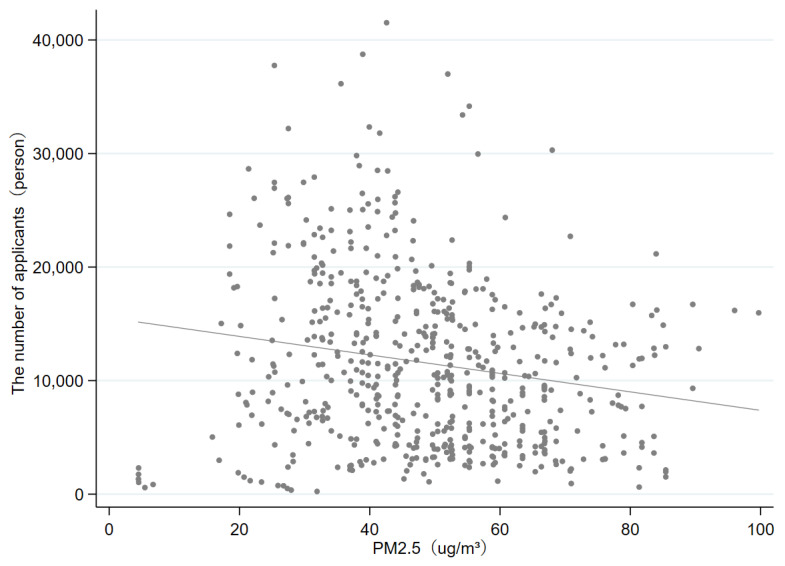
PM_2.5_ concentration and the number of applicants. Note: This figure displays the relationship between PM_2.5_ concentration and the number of applicants for all sample universities.

Before reporting the benchmark results, we preliminarily examine the relevance between PM_2.5_ concentration and the number of applicants. From the fitting diagram (Figure 2), there is a strong negative correlation between PM_2.5_ concentration and the quantity of applicants. In addition, the regression coefficient is −236.6853, and *p*-value is 0.00, which is significant at 1% level. It illustrates that the more severe the city air pollution, the fewer the number of applicants. Next, we will use a panel fixed-effect model to empirically test the specific impacts of air pollution on the applicants’ university-city choices.

### 2.3. Empirical Strategy

Theoretically, we use Equation (1) to estimate the impacts of air pollution on the university-city choices of applicants.

(1) $$Apply_{icpt}=\alpha_{0}+\alpha_{1}PM_{2.5icpt}+\alpha_{2}X_{icpt}+\lambda_{i}+\pi_{t}+\varepsilon_{icpt}$$

In Equation (1), $Apply_{icpt}$ denotes the number of applicants for UNGEE in each university; $PM_{2.5icpt}$ is the annually average PM_2.5_ concentration of the city where the intended university is located; $X_{icpt}$ is a series of control variables; and $\lambda_{i}$ represents the fixed effect of a school individual, which can control for time-invariant confounders specific to school; $\pi_{t}$ is a time fixed effect, which can account for shocks common on all universities and cities in a particular year; and $\varepsilon_{icpt}$ denotes the random error. In each variable, the subscript i denotes university, *c* means city, *p* is province, and t represents year. The $\alpha_{1}$ depicts the average impacts of air pollution on the number of applicants in each university. If its estimated value is significantly negative, it means that air pollution has a negative impact on the quantity of applicants. Its amount indicates that the average PM_2.5_ concentration changes by 1 ug/m^3^, how many will change in the number of applicants in each university.

Based on this, this paper may meet endogenous challenges caused by omitted variables, measurement error, and reverse causation. The dependent variable of this paper measures applicants’ choosing intentions rather than actual migration behaviors, so there is no need to consider the endogenous problems caused by simultaneity. In terms of omitted variables, although Equation (1) controls the fixed effect of university and avoids the omitted variables at schools, the factors affecting applicants’ university-city selection at the city and province are more complex, and it is unfeasible to fully involve in the model, so there will exist unconsidered factors in the error term. Additionally, the data that we manually collected may exist with a measurement error. To overcome the challenges, we use the instrumental variable of air pollution to perform a two-stage least squares (2SLS) estimation based on Equations (2) and (3).

The first stage:

(2) $$PM_{2.5icpt}=\beta_{0}+\beta_{1}Avgwindsp_{icpt}+\beta_{2}X_{icpt}+\lambda_{i}+\pi_{t}+\mu_{icpt}$$

The second stage:

(3) $$Apply_{icpt}=\gamma_{0}+\gamma_{1}P{\overset{⌢}{M}}_{2.5icpt}+\gamma_{2}X_{icpt}+\lambda_{i}+\pi_{t}+\nu_{icpt}$$

**Figure 3:**
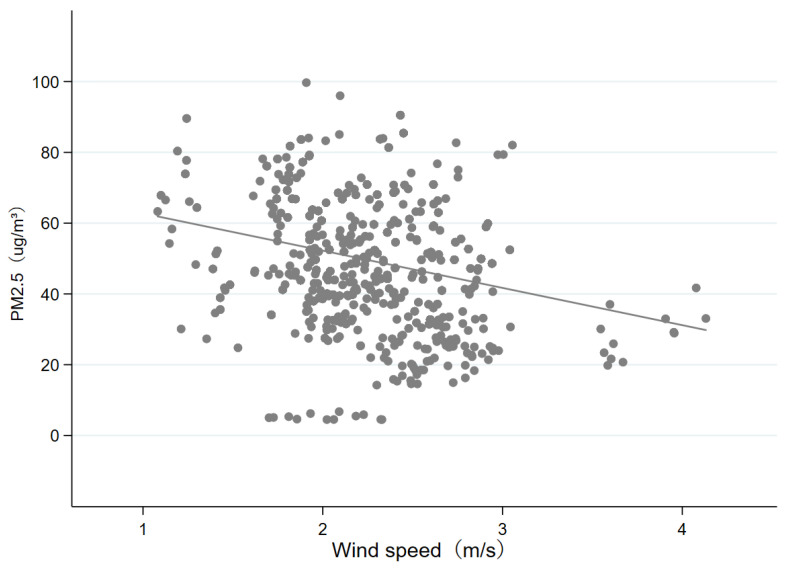
Wind speed and PM_2.5_ concentration. Note: This figure displays the relationship between annually average wind speed and the PM_2.5_ concentration of all sample cities.

To determine the instrumental variable, we mainly consider whether the selection of instrumental variable meets the restriction of relevance and exclusion restriction at the same time. The relevance restriction means that the instrumental variable has a clear effect on the endogenous variable (PM_2.5_), and the exclusion restriction means that the only reason for the relationship between the instrument variable and dependent variable is the endogenous variable. The existing literature usually chooses meteorological indicators as the instrumental variable for air pollution. For example, based on the fact that the higher the altitude, the higher the atmospheric temperature will be, Arceo et al. (2016) and Chen et al. (2022) use thermal inversion as the instrumental variable of air pollution. Normally, the hot air rises and the cold air falls, which facilitate the diffusion of pollutants, but in turn, there is a thermal inversion, and the pollutants are not easy to diffuse. So, the more serious the thermal inversion, the more severe the air pollution. When studying the impacts of environmental regulation on the layout of polluting industries, based on the fact that the intensity of regulation is related to the degree of pollution, Broner et al. (2012) use the ventilation coefficient as the instrumental variable for environmental regulation [25]. Generally, the higher the wind speed, the lighter the pollution will be, and the better the condition of lateral diffusion. So, the smaller the ventilation index, the stronger the environmental regulation. Similar to this, we choose the annually average wind speed (Avgwindsp) as the instrumental variable of air pollution. In general, if the wind speed is higher, the lateral diffusion conditions of pollutants will be better, and the PM_2.5_ concentration will be lower. Therefore, the PM_2.5_ concentration and wind speed will have a negative relationship. Figure 3 depicts the fitting scatter diagram of the instrumental variable (annually average wind speed) and endogenous variable (PM_2.5_ concentration). The regression coefficient is −10.5289, and the *p*-value is 0.00, which is significant at 1% level. It discloses that two variables are changing conversely, which satisfies the relevance restriction, and the wind speed is usually determined by the city’s meteorological conditions, which is not directly related to other factors that affect applicants’ university-city selection, so it also meets the exclusion restriction.

## 3. Empirical Results

### 3.1. The Baseline Results

As a reference, the ordinary least squares (OLS) estimation is first performed based on Equation (1), and the variables of university, urban, and climate are added to the equation step by step. The columns (1)–(3) of Table 2 show the regression results.

Column (1) is the estimated result of adding only the variable of university. The estimated coefficient of PM_2.5_ is significantly negative, and the estimated coefficient of the number of students to be enrolled is significantly positive. Columns (2) and (3) are the regression results of adding the variables of city and climate in turn. From the column (3) that adds all the control variables, the estimated coefficient of PM_2.5_ is still significantly negative, revealing that the PM_2.5_ concentration has a negative impact on the university-city selection of applicants. The higher the PM_2.5_ concentration in the city where the aimed at university is located, the smaller the number of applicants to the university will be. The estimated coefficient of the number of students to be enrolled is still significantly positive at the 1% level, demonstrating that the number of applicants will increase with the increase of students’ number to be enrolled. From the perspective of the control variables, the estimated coefficient of the consumer price index (CPI) is significantly positive, which is inconsistent with our expectation, and the estimated coefficient of population density is significantly negative, indicating that the applicants may dislike a city of high population density. Lastly, the estimated coefficients of the annually average temperature and annually average rainfall are significantly negative, showing that the applicants may escape from the city of high temperature and rainy climate.

Next, we use the annually average wind speed as an instrumental variable of air pollution to estimate Equations (2) and (3). To be consistent with the OLS estimation, the variables of university, city, and climate are added to the equations in turn, and the estimation results are shown in columns (1)–(3) of Table 3.

From column (1), the first stage shows that the estimated coefficient of average wind speed (Avgwindsp) is significantly negative at the 1% level, indicating that the faster the wind speed, the lower the PM_2.5_ concentration will be. And the F value (41.18) is larger than 10, there is no risk of a weak instrumental variable. In the second stage, the estimated coefficient of PM_2.5_ is significantly negative at the 5% level but compared with the result estimated by the ordinary least squares (OLS) (column 1 of Table 2), the absolute value becomes larger, revealing that the OLS estimated result could be underestimated. Columns (2) and (3) are the estimated results of adding urban and climatic variables in turn. From column (3), the coefficient of PM_2.5_ is still significantly negative, indicating that the increase in PM_2.5_ concentration by an average 1 ug/m^3^ will generate a reduction of applicants’ quantity by about 250, which accounts for 2.2% of the average quantity of all applicants. The estimated coefficient of the number of students to be enrolled is still significantly positive; specifically, the number of students to be enrolled increases by 1, and the amount of applicants increases by around 2. Additionally, the estimated coefficients of population density, annually average temperature, and annually average rainfall are significantly negative too. One exception is the consumer price index (CPI), although the result is still positive, but it is no longer significant, demonstrating that the consumer price index (CPI) may have few causal effects on applicants’ university-city choices. Meanwhile, the first stage shows that the estimated coefficient of average wind speed is still significantly negative, and the F value (30.77) is still higher than 10, further certifying that our determination of the instrumental variable is plausible.

Due to different majors, the attention to the air information of applicants for different disciplines may be differential, and the ratio of gender between different disciplines are also distinguishing. Existing studies have shown that compared with males, females are more concerned about air quality [26,27]. Taking the difference into consideration, we further investigate the effect of air pollution on different subjects’ applicants. The estimation result is shown in columns (1) and (2) of Table 4.

Column (1) is the estimated result of social science. The estimated coefficient of PM_2.5_ is −200.0694, which means that the average PM_2.5_ concentration increases for 1 ug/m^3^, and the number of applicants for social science decreases by about 200. Column (2) is the estimated result of natural science: for every 1 ug/m^3^ increase in PM_2.5_ concentration, the number of applicants for natural science will decrease by about 50, but it is statistically insignificant, demonstrating that air pollution may have few significant effects on the choices of natural science applicants. It is consistent with our anticipation that the applicants of social science may pay more attention to air quality and have a higher standard for life, due to the professional knowledge that they are exposed to every day. In addition, social science has a larger scale of female students who are more sensitive to air pollution than male students, so the sensitivity to it is higher.

### 3.2. Robustness Checks

In order to further verify the robustness of benchmark regression results, we mainly consider replacing the independent variable, control variable, and excluding other affecting factors to test the results.

#### 3.2.1. Alternative Air Pollution Indicators

China’s outdoor pollutants of air pollution mainly include PM_2.5_ and SO_2_ [28,29,30]. SO_2_ is easily soluble in human body fluids, and the long-run existence can lead to upper respiratory tract infections, chronic bronchitis, emphysema, and other diseases, so it may also affect the university-city choices of applicants. We substitute PM_2.5_ with SO_2_, re-estimate Equations (2) and (3), and the regression result is shown in column (2) of Table 5.

The result shows that the estimated coefficient of SO_2_ is negative at the 10% level; a rise in SO_2_ concentration by one unit on average causes a reduction of applicants’ numbers by about 57. It further demonstrates that air pollution does have a negative impact on the university-city choices of applicants, which verifies the robustness of the benchmark regression results.

#### 3.2.2. Alternative University Control Variable

In the baseline regression, we choose the number of students to be enrolled that published in their intended university’s admission brochure as the university control variable. However, when applying for UNGEE, applicants also focus on the admission ratio of recent years, as the admission proportion can reflect the competition and difficulty of target university. In view of this, we displace the number of students to be recruited with one year lag of admission ratio to re-regress Equations (2) and (3); the result is reported in column (3) of Table 5. It shows that the estimated coefficient of PM_2.5_ is −215.1581 at a 5% significant level, which is analogous to the estimated coefficient of benchmark regression (−250.4680), indicating that the baseline regression result is stable.

#### 3.2.3. Excluding the Agglomeration of Double First-Class Universities

Some double first-class universities exist as an agglomeration in partial Chinese cities; the agglomeration in a single city may underestimate the impact of air pollution on applicants’ university-city choices. During the data collection process, we notice that Beijing, Shanghai, Wuhan, Nanjing, and other traditional first-tier and second-tier cities have more than 5 double first-class universities. In order to eliminate the impact, we exclude cities with more than 5 double first-class universities in the sample and re-regress the equations. Column (4) of Table 5 reports the regression result. After moving partial cities, although the absolute value of the estimated coefficient in PM_2.5_ becomes larger, it is still significantly negative, showing that the benchmark regression result is steady. Specifically, for every 1 ug/m^3^ increase in PM_2.5_ concentration on average, the number of applicants applying for UNGEE decreases by about 526, which is greater than the benchmark result (250). The reason is that these excluding cities such as Beijing, Shanghai, Wuhan, Nanjing, and so on are the traditional preferred city for study. Furthermore, the gathering of double first-class universities represents more abundant educational resources, which can bring more opportunities and growth to applicants, and may increase the attractiveness to applicants. Thereby, these advantages can weaken the negative impact of air pollution on applicants’ choosing intentions. Therefore, after removing these sample cities, the regression coefficient becomes larger.

#### 3.2.4. Considering the Non-Double First-Class Universities

The sample that we use in the baseline regression is the data of 76 double first-class universities. During the data collection process, we note that the official websites of some non-double first-class universities have also published application data over these years. Therefore, we add the data that we manually crawl from the website of 82 non-double first-class universities into the sample to re-regress the equations, and the result is shown in column (5) of Table 5. It shows that the coefficient of PM_2.5_ is significantly negative, so the benchmark conclusion still holds. For every 1 ug/m^3^ increase in PM_2.5_ concentration on average, the number of applicants decreases by about 136. Compared with the benchmark result (250), the absolute value decreases, illustrating that the sample of non-double first-class universities lowers the sensitivity. Generally speaking, applicants who apply for non-double first-class universities usually are the group that did not perform very well in gaokao and usually have a relatively poor starting point during their undergraduate degree, so the range of universities they can choose from is limited. Therefore, compared with air quality, they may pay more attention to other factors such as who is more likely to be admitted when make their university-city choices.

## 4. The Heterogeneous Effects

In addition to the average effects, we further investigate how the effects differ in terms of university. Firstly, according to the results of China’s fourth round of subject evaluation, we divide the universities into different qualities, and investigate the heterogeneity between world-class universities and first-class discipline universities. Secondly, according to the division of university by the MOE of PRC, the heterogeneity between comprehensive and non-comprehensive universities is examined. Finally, we take the location of universities into consideration, and we estimate the heterogeneity between northern and southern universities.

### 4.1. Different Quality Universities

The attractiveness of a different quality university for applicants has certain differences. Compared with first-class discipline universities, world-class universities are more likely to be pursued by applicants. Therefore, the air pollution sensitivity between applicants who apply for different quality universities may be distinguishing. China’s fourth round of subject evaluation divided double first-class universities into world-class universities and first-class discipline universities. Generally speaking, world-class universities usually have a better quality than first-class discipline universities. According to the result, we separate 76 universities into world-class universities and first-class discipline universities and examine the heterogeneity between them. We define the dummy variable for the world-class university and its interaction with the air pollution measure in Equations (2) and (3) to estimate the heterogeneous effect. The regression result is shown in column (1) of Table 6.

The result reveals that air pollution has a significantly negative impact on applicants for double first-class universities. However, compared with first-class discipline universities, the world-class universities lower the sensitivity of air pollution for applicants. It demonstrates that air pollution indeed affects the choices of applicants, but the quality of the university can weaken the effect. The world-class universities are those at the top in China, which are also the targets that all students pursue, so applicants will only take air pollution a little into account when faced with a better-quality university choice. The result is similar with our conclusion in the robustness checks of excluding the agglomeration of double first-class universities, which further illustrates the potential importance of university quality and educational resources in the university-city selection of applicants.

### 4.2. Different Types of Universities

The university of a different type contains different majors, and comprehensive universities usually contain more sorts of majors than non-comprehensive universities. So, the number of applicants who apply for comprehensive universities may be larger than non-comprehensive universities, and there may be differences in air pollution sensitivity among them. Based on this, according to the sort of university from the MOE of PRC (Search each university in https://www.baidu.com/, accessed on 26 December 2020), we divide 76 double first-class universities into comprehensive and non-comprehensive, and then define the dummy variable for the comprehensive university and its interaction with the air pollution measure in Equations (2) and (3) to estimate the heterogeneous effect. The estimated result is presented in column (2) of Table 6. The result discloses that air pollution has a significantly negative impact on the choices of applicants, but there is no significant difference in applicants for the two types of universities. It demonstrates that there is no significant difference in the sensitivity of applicants to air pollution among different types of universities.

### 4.3. Different Regions of Universities

The Qinling-Huaihe geographical line divides China into the two parts of the south and north. Due to the cold climate in winter, the Chinese government provides a free heating system for all households and indoor places in the north, and the supply of heating requires a large amount of fossil fuels such as coal, which emits a lot of pollutants, but it does not exist in the south. The policy difference causes an initial difference in air pollution between the two regions [3,7]. In view of this, we divide 76 double first-class universities into southern and northern according to the Qinling-Huaihe boundary, and then define the dummy variable for northern university and its interaction with air pollution measure in Equations (2) and (3) to estimate the heterogeneous effect. Column (3) of Table 6 represents the regression result.

The result shows that air pollution has little impact on applicants for southern universities, but the universities of the north significantly aggravate the negative effect on applicants, which is consistent with our anticipation. The applicants who live or study in China have a full recognition that air pollution in the north (the average PM_2.5_ concentration is 51.41 ug/m^3^) is more serious than in the south (the average PM_2.5_ concentration is 47.99 ug/m^3^). Worse air quality may affect their physical and mental health during the study period for a master’s degree, and the inherent awareness may drive them to evade a northern university when other reference factors such as university quality are identical.

**Figure 4:**
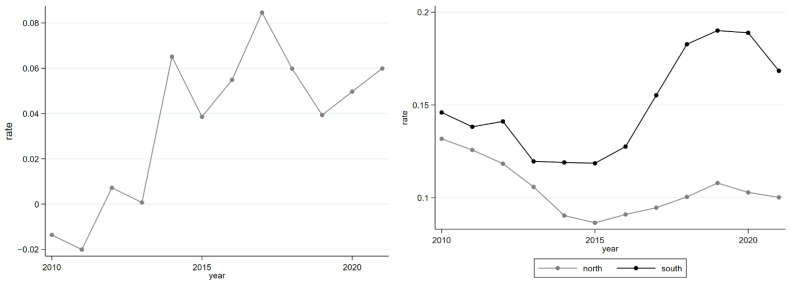
Northern PM_2.5_ concentration ratio and northern-southern ratio of applicants’ number. Note: The (**left**) figure displays the ratio that northern PM_2.5_ concentrations are higher than all of the country, and the (**right**) figure displays the ratio that northern and southern applicants’ number account for the whole country.

To verify the above conclusion of different regions, we analyze the application data and PM_2.5_ concentration data and then draw the trend graphs (Figure 4). The left side of Figure 4 shows the trend of northern air pollution deterioration ratio (deterioration ratio = (average northern PM_2.5_ concentration − average national PM_2.5_ concentration)/average national PM_2.5_ concentration), and the right side of Figure 4 is the trend of the proportion that the applicants of northern and southern universities account for the whole country from 2010 to 2021. From the left side, we can find that the ratio has gradually increased during the period, manifesting that compared with the whole country, northern air pollution is aggravating. From the right, the proportion of the number of applicants to northern universities is gradually decreasing, while the ratio of southern universities is gradually increasing, summarizing that with the deterioration of northern air quality, the university-city choosing intentions of applicants are shifting from north to south. The factual analysis confirms our above conclusion reasonably.

## 5. Comparison with Existing Literature

Ultimately, we summarize the estimates of the effects of air pollution on migration or migration intention in the existing literature in Table 7. Firstly, some literature focuses topics on talents [13,14,31]. Based on the same perspective of college or university graduates and the same pollutant of air pollution as us, Lai et al. (2021) find that with a 1 ug/m^3^ increase in PM_2.5_ concentration in the original city, the probability of college graduates to leave the city increases by 1%, which is lower than our result (2.17%). From the view of corporations, Wang and Wu (2021) disclose that with a 1 ug/m^3^ increase in PM_2.5_ concentration in the original city, the stock of technologically innovative professionals decreases by 0.15%. Using AQI as a pollutant of air pollution, Xue et al. (2021) conclude that with a 100-point increase in AQI in a destination city, the search volume index of choosing a destination city as the intended work place for corporate human capital decreases 2.74%, and the magnitude is similar with our result (2.17%).

In this paper, we use the application of UNGEE to measure migration intention, and some existing literature also measures migration intention from other perspectives. Treating the Baidu search index on “emigration” as the migration intention, Qin and Zhu (2018) reveal that the increase of 100-point in the AQI of the destination city decreases 2.3–4.8% emigration interests to the destination city, which is lower than the urban or floating migration interest in settlement [32,33]. Apart from migration intention, some literature also pay attention to actual migration. Liu and Yu (2020) and Chen et al. (2022) disclose that the net outflow of population increases 15.1% and 0.53% with the increase of 100-point in AQI and 1 ug/m^3^ in the PM_2.5_ concentration of the original city. Lastly, using children migration data, Li et al. (2020) detect that the probability of children migration with parents decreases 5.18% with a 1 ug/m^3^ increase in the PM_2.5_ concentration in the destination city [34]. Based on the same pollutant of air pollution, the result is larger than ours, which reveals that the children who are identified as the vulnerable group are more sensitive to air pollution than teenagers.

## 6. Conclusions

Based on the merging of application data for UNGEE of 76 double first-class universities and urban PM_2.5_ concentration data in China, this paper uses wind speed as the instrumental variable of air pollution and exploits the two-stage least squares (2SLS) method to empirically investigate the effect of air pollution on applicants’ university-city choices. We find that air pollution has a negative impact on the university-city choices of applicants: the average increase in urban PM_2.5_ concentration by 1 ug/m^3^ reduces the number of applicants for each university of a destination city by about 250. Among them, the number of applicants for social science decreases significantly by 200, and the number of applicants for natural science decreases by 50. Compared with previous research, our conclusions are subtly different. Additionally, the impacts of air pollution on applicants are highly heterogeneous. Air pollution has more significantly negative effects on applicants of first-class discipline universities than world-class universities. The reason is that the quality of university can weaken the negative effects of air pollution on applicants. However, the impacts have no significant difference in comprehensive and non-comprehensive universities. Lastly, due to the reason that air pollution in the north is more deteriorated than in the south, the universities located in the north aggravate the negative effects of air pollution on applicants, which results in the fact that university-city selecting intentions of applicants have a tendency that shift from north to south.

This paper provides evidence for studies on the effects of air pollution on high human capital migration intention from developing countries. Furthermore, by comparing with previous studies, we disclose a differential completion from the perspective of university. The conclusions of this paper show that applicants who apply for UNGEE are highly sensitive to air pollution, and the applicants of social science are more sensitive than natural science. There are also differences between different qualities and regions of the universities. All of the above conclusions can be further extended to the working location decisions of graduates, or flowing orientation of talents, and provide practical recommendations for the introduction of talents in a number of cities. In the mature development stage, governments should prioritize economic development, but also try to trade off the environment condition, and take measures to build an “environmentally friendly, resource-saving, and livable” city to attract a “knowledge-based and innovative” labor force to join in. In addition, taking heterogeneous effects into consideration, policy makers should implement differentiated policies for different groups and regions.

This study also has some limitations: the first is that we do not have more microscopic individual data, such as personal information for each applicant, which can track the migration origin and destination of each applicant. We can only use the quantity of applicants for every university to measure migration intentions. The second is that we do not have the physical and mental health data of the Chinese university students at our sample universities, so we cannot analyze the mechanism of impact from physical and mental health channels. And so we can’t know that how air pollution affects applicants’ university-city choices.

## Figures and Tables

**Table 1:** Descriptive statistics of main variables.

Variables		Obs	Mean	SD	Min	Max
Apply		585	11,547	7446	237	41,522
Rw_apply	Social science	585	6654	5654	6	28,297
Zr_apply	Natural science	585	4895	4097	0	21,627
PM_2.5_		585	49.79	16.73	4.50	99.71
Enrollment		585	2809	1584	145	8737
Rj_gdp		585	133.73	64.84	16.91	462.95
Industry 3_rate		585	59.72	13.06	25.69	83.87
CPI		585	137.34	13.38	109.65	171.37
Popu		585	957.61	646.68	15.61	4119
Edu_cost		585	1573	2012	14.13	9623
Transpor		585	15.29	7.68	1.27	167.70
Avgtemp		585	14.49	4.05	3.10	25.53
Avgrain		585	26.57	13.96	4.03	71.97
Avgwindsp		585	2.23	0.41	1.08	4.13

Note: The consumer price index (CPI) is calculated with 100 based on 2008.

**Table 2:** Impact of air pollution on applicants’ number (OLS estimates).

Variables	OLS		
Apply	(1)	(2)	(3)
PM_2.5_	−58.5446 **	−58.6956 **	−56.5464 **
	(23.10)	(23.82)	(23.71)
Enrollment	2.0565 ***	2.1590 ***	2.1918 ***
	(0.26)	(0.26)	(0.26)
Rj_gdp		−12.4473	−10.0484
		(8.94)	(9.95)
Industry_3rate		−49.3432	−42.9970
		(57.23)	(56.97)
CPI		296.9077 **	293.5499 **
		(126.15)	(126.12)
Popu		−0.8787 **	−0.9502 **
		(0.45)	(0.45)
Transpor		8.6109	−5.2994
		(21.06)	(21.90)
Edu_cost		0.1588	0.1245
		(0.13)	(0.13)
Avgtemp			−906.4734 **
			(396.44)
Avgrain			−53.6604 **
			(24.49)
School FE	Yes	Yes	Yes
Year FE	Yes	Yes	Yes
N	585	585	585
R^2^	0.5340	0.5396	0.5453

Note: Standard errors clustered at city level are reported in the brackets. *** *p* < 0.01, ** *p* < 0.05.

**Table 3:** Impact of air pollution on applicants’ number (2SLS estimates).

Second Stage			
Variables	2SLS		
Apply	(1)	(2)	(3)
PM_2.5_	−250.4749 **	−324.9521 ***	−250.4680 **
	(104.96)	(103.45)	(97.61)
Enrollment	2.2718 ***	2.5286 ***	2.4464 ***
	(0.30)	(0.33)	(0.31)
Rj_gdp		1.7623	−0.2087
		(11.35)	(10.68)
Industry_3rate		−115.8101 *	−90.9361
		(68.81)	(65.07)
CPI		153.2544	194.7881
		(151.26)	(142.80)
Popu		−1.4972 ***	−1.3889 ***
		(0.55)	(0.52)
Edu_cost		0.0296	0.0336
		(0.16)	(0.15)
Transpor		13.6354	1.2228
		(23.67)	(23.57)
Avgtemp			−733.7366 *
			(431.00)
Avgrain			−54.7615 **
			(26.12)
School FE	Yes	Yes	Yes
Year FE	Yes	Yes	Yes
N	585	585	585
R^2^	0.5491	0.5152	0.5680
**First Stage**			
Avgwindsp	−7.4706 ***	−8.7096 ***	−9.2802 ***
	(1.39)	(1.47)	(1.57)
F	41.18	35.22	30.77
N	585	585	585

Note: Standard errors clustered at city level are reported in the brackets. *** *p* < 0.01, ** *p* < 0.05, * *p* < 0.1.

**Table 4:** Different discipline applicants.

Second Stage		
	2SLS	
Variables	(1)	(2)
Apply	Social Science	Natural Science
PM_2.5_	−200.0694 ***	−50.1722
	(73.80)	(44.07)
University	Yes	Yes
Urban	Yes	Yes
Climate	Yes	Yes
School FE	Yes	Yes
Year FE	Yes	Yes
N	585	585
R^2^	0.4359	0.5507
**First Stage**		
Avgwindsp	−9.2802 ***	−9.2802 ***
	(1.57)	(1.57)
F	30.77	30.77
N	585	585

Note: Standard errors clustered at city level are reported in the brackets. University control includes number of students to be recruited; urban controls include GDP per capita, the proportion of the tertiary industry, consumer price index, population density, education expenditure per capita, number of buses and trams (per 10,000 people); climate controls include annually average temperature, annually average rainfall. *** *p* < 0.01.

**Table 5:** Robustness check results.

		Second Stage			
	2SLS				
Variables	(1)	(2)	(3)	(4)	(5)
Apply	Baseline	Replace Independent Variable	Replace University Control Variable	Exclude Agglomeration of Double First-Class Universities	Add Non-Double First-Class Universities
PM_2.5_	−250.4680 **		−215.1581 **	−526.1274 **	−135.2575 *
	(97.61)		(97.44)	(190.00)	(73.75)
SO_2_		−57.2924 *			
		(30.24)			
University	Yes	Yes	Yes	Yes	Yes
Urban	Yes	Yes	Yes	Yes	Yes
Climate	Yes	Yes	Yes	Yes	Yes
School FE	Yes	Yes	Yes	Yes	Yes
Year FE	Yes	Yes	Yes	Yes	Yes
N	585	585	585	288	1117
R^2^	0.5680	0.2077	0.5780	0.4578	0.5679
		**First Stage**			
Avgwindsp		−40.5707 **	−9.1943 ***	−11.4495 ***	−6.9300 ***
		(15.90)	(1.57)	(3.00)	(1.23)
F		16.30	30.82	28.64	30.64
N		585	585	288	1117

Note: Standard errors clustered at city level are reported in the brackets. University control of column (3) includes ratio of enrollment, university control of other columns includes number of students to be recruited; urban controls include GDP per capita, the proportion of the tertiary industry, consumer price index, population density, education expenditure per capita, number of buses and trams (per 10,000 people); climate controls include annually average temperature, annually average rainfall. *** *p* < 0.01, ** *p* < 0.05, * *p* < 0.1.

**Table 6:** The heterogeneous effects on migration intentions.

Second Stage			
	2SLS		
Variables	(1)	(2)	(3)
Apply			
PM_2.5_	−431.7390 **	−404.8751 *	−33.6891
	(196.27)	(218.04)	(132.13)
${\mathrm{PM}}_{2.5}\;\times$ World-class university	420.1479 **		
	(189.89)		
${\mathrm{PM}}_{2.5}\;\times$ Comprehensive university		348.2737	
		(336.12)	
${\mathrm{PM}}_{2.5}\;\times$ Northern university			−186.2481 *
			(98.38)
University	Yes	Yes	Yes
Urban	Yes	Yes	Yes
Climate	Yes	Yes	Yes
School FE	Yes	Yes	Yes
Year FE	Yes	Yes	Yes
N	585	585	585
R^2^	0.3631	0.3281	0.5856
**First Stage**			
Avgwindsp	−6.2735 ***	−11.1137 ***	−5.4436 ***
	(1.84)	(1.91)	(1.40)
F	29.52	30.42	29.68
N	585	585	585

Note: Standard errors clustered at city level are reported in the brackets. University control includes number of students to be recruited; urban controls include GDP per capita, the proportion of the tertiary industry, consumer price index, population density, education expenditure per capita, number of buses and trams (per 10,000 people); climate controls include annually average temperature, annually average rainfall. *** *p* < 0.01, ** *p* < 0.05, * *p* < 0.1.

**Table 7:** Summary of the estimated air pollution impacts on migration or migration intention in existing literature.

Paper	Country	Type of Migration	Pollutants	Factor of Dependent Variable	Increase in Pollutant Concentration	Change in Migration or Migration Intention
This study	China	Applicants of UNGEE	PM_2.5_	The quantity of applicants (move into destinational city)	1 ug/m^3^ (destinational city)	−2.17%
Qin and Zhu (2018)	China	Emigration interests in prefecture cities	AQI	Baidu search index on “emigration” (move into destinational city)	100-point (destinational city)	−2.30–4.80%
Li et al. (2020)	China	Children’s migration	PM_2.5_	Probability of children’s migration with parents (move into destinational city)	1 ug/m^3^ (destinational city)	−5.18%
Liu and Yu (2020)	China	Urban migration	AQI	Migrants’ interest in settling down in current city (stay in original city)	100-point (original city)	−15.1%
Lai et al. (2021)	China	College graduates	PM_2.5_	Probability to leave current city (move out of original city)	1 ug/m^3^ (original city)	+1.00%
Wang and Wu (2021)	China and India	Technological innovative professionals (TIP)	PM_2.5_	Stock of TIP (move out of original city)	1 ug/m^3^ (original city)	+0.15%
Jia and Chen (2021)	China	Floating migrants	AQI	Migrants’ settlement intentions (move into destinational city)	100-point (destinational city)	−33.2%
Xue et al. (2021)	China	Corporate human capital (executives)	AQI	Search volume index of intended work places (move into destinational city)	100-point (destinational city)	−2.74%
Chen et al. (2022)	China	Migration in China’s counties	PM_2.5_	Net-outmigration ratio (move out of original city)	1ug/m^3^ (original city)	+0.53%

## Data Availability

No new data were created or analyzed in this study. Data sharing is not applicable to this article.

## Appendix A

**Table A1:** List of 76 double first-class universities.

Beijing (15)	Beijing University, Renmin University of China, Beijing University of Aeronautics and Astronautics, China Agricultural University, Beijing Normal University, Minzu University of China, Central University of Finance and Economics, University of International Business and Economics, China University of Political Science and Law, Beijing University of Posts and Telecommunications, Beijing Forestry University, Capital Normal University, Beijing Foreign Studies University, China University of Mining & Technology (Beijing), University of Chinese Academy of Sciences
Tianjin (4)	Nankai University, Hebei University of Technology, Tianjin Medical University, Tiangong University
Shanghai (7)	Tongji University, Shanghai Jiao Tong University, East China Normal University, Donghua University, Shanghai International Studies University, Shanghai University of Finance and Economics, Shanghai University
Chongqing (1)	Southwest University
Guangzhou (4)	Sun Yat-sen University, South China University of Technology, Jinan University, South China Normal University
Nanjing (5)	Nanjing University, Southeast University, Nanjing University of Posts and Telecommunications, Nanjing Forestry University, Nanjing Agricultural University
Wuxi (1)	Jiangnan University
Suzhou (1)	Soochow University
Xuzhou (1)	China University of Mining and Technology (Xuzhou)
Hangzhou (1)	Zhejiang University
Ningbo (1)	Ningbo University
Wuhan (5)	Wuhan University, China University of Geosciences (Wuhan), Huazhong Agricultural University, Zhongnan University of Economics and Law, Central China Normal University
Changsha (3)	Central South University, Hunan University, National University of Defense Technology
Hefei (1)	Anhui University
Fuzhou (1)	Fuzhou University
Xiamen (1)	Xiamen University
Zhengzhou (1)	Zhengzhou University
Kunming (1)	Yunnan University
Shihezi (1)	Shihezi University
Tibet (1)	Tibet University
Xian (4)	Xidian University, Shaanxi Normal University, Northwest University, Chang’an University
Xianyang (1)	Northwest A&F University
Chengdu (2)	Sichuan University, Southwestern University of Finance and Economics
Dalian (1)	Dalian University of Technology
Shenyang (2)	Northeastern University (Shenyang), Liaoning University
Harbin (1)	Harbin Engineering University
Haikou (1)	Hainan University
Yinchuan (1)	Ningxia University
Lanzhou (1)	Lanzhou University
Qinhuangdao (1)	Northeastern University (Qinhuangdao)
Jinan (1)	Shandong University (Jinan)
Weihai (1)	Shandong University (Weihai)
Qingdao (2)	Ocean University of China, China University of Petroleum (East China)
Inner mongolia (1)	Inner Mongolia University

## References

[1] Schlenker W., Walker W.R. (2015). Airports, air pollution, and contemporaneous health. Rev. Econ. Stud..

[2] Arceo E., Hanna R., Oliva P. (2016). Does the Effect of Pollution on Infant Mortality Differ between Developing and Developed Countries? Evidence from Mexico City. Econ. J..

[3] Ebenstein A., Fan M., Greenstone M., He G., Zhou M. (2017). New Evidence on the Impact of Sustained Exposure to Air Pollution on Life Expectancy from China’s Huai River Policy. Proc. Natl. Acad. Sci. USA.

[4] He G., Liu T., Zhou M. (2020). Straw burning, PM2. 5, and death: Evidence from China. J. Dev. Econ..

[5] Fan M., He G., Zhou M. (2020). The winter choke: Coal-fired heating, air pollution, and mortality in China. J. Health Econ..

[6] Deschenes O., Wang H., Wang S., Zhang P. (2020). The effect of air pollution on body weight and obesity: Evidence from China. J. Dev. Econ..

[7] Chen Y., Ebenstein A., Greenstone M., Li H. (2013). Evidence on the Impact of Sustained Exposure to Air Pollution on Life Expectancy from China’s Huai River Policy. Proc. Natl. Acad. Sci. USA.

[8] Zhang X., Zhang X., Chen X. (2017). Happiness in the air: How does a dirty sky affect mental health and subjective well-being?. J. Environ. Econ. Manag..

[9] Zhang Z., Hao Y., Mu Z.N. (2018). Does environmental pollution affect labor supply? An empirical analysis based on 112 cities in China. J. Clean. Prod..

[10] Ito K., Zhang S. (2020). Willingness to pay for clean air: Evidence from air purifier markets in China. J. Political Econ..

[11] Chen S., Oliva P., Zhang P. (2022). The Effect of Air Pollution on Migration: Evidence from China. J. Dev. Econ..

[12] Zheng S., Zhang X., Sun W., Lin C. (2019). Air Pollution and Elite College Graduates’ Job Location Choice: Evidence from China. Ann. Reg. Sci..

[13] Xue S., Zhang B., Zhao X. (2021). Brain Drain: The Impact of Air Pollution on firm Performance. J. Environ. Econ. Manag..

[14] Lai W., Song H., Wang C., Wang H. (2021). Air pollution and brain drain: Evidence from college graduates in China. China Econ. Rev..

[15] Clark W.A., Ledwith V. (2007). How much does income matter in neighborhood choice?. Popul. Res. Policy Rev..

[16] So K.S., Orazem P.F., Otto D.M. (2001). The effects of housing prices, wages, and commuting time on joint residential and job location choices. Am. J. Agric. Econ..

[17] Wozniak A. (2010). Are college graduates more responsive to distant labor market opportunities?. J. Hum. Resour..

[18] Plantinga A.J., Détang-Dessendre C., Hunt G.L., Piguet V. (2013). Housing prices and inter-urban migration. Reg. Sci. Urban Econ..

[19] Qin Y., Zhu H. (2018). Run away? Air Pollution and Emigration Interests in China. J. Popul. Econ..

[20] Fu S., Viard V.B., Zhang P. (2021). Air Pollution and Manufacturing Firm Productivity: Nationwide Estimates for China. Econ. J..

[21] Zivin J.G., Liu T., Song Y., Tang Q., Zhang P. (2020). The unintended impacts of agricultural fires: Human capital in China. J. Dev. Econ..

[22] Burkhardt J., Bayham J., Wilson A., Carter E., Berman J.D., O’Dell K., Pierce J.R. (2019). The effect of pollution on crime: Evidence from data on particulate matter and ozone. J. Environ. Econ. Manag..

[23] Chen C., Li C., Li Y., Liu J., Meng C., Han J., Xu D. (2018). Short-term effects of ambient air pollution exposure on lung function: A longitudinal study among healthy primary school children in China. Sci. Total Environ..

[24] Shepherd A., Mullins J.T. (2019). Arthritis diagnosis and early-life exposure to air pollution. Environ. Pollut..

[25] Broner F., Bustos P., Carvalho V.M. Sources of Comparative Advantage in Polluting Industries. NBER Working Paper Series. 2012. No.18337. https://www.nber.org/papers/w18337.

[26] Arias-Ortiz N.E., Icaza-Noguera G., Ruiz-Rudolph P. (2018). Thyroid Cancer Incidence in Women and Proximity to Industrial Air Pollution Sources: A Spatial Analysis in a Middle Size City in Colombia. Atmos. Pollut. Res..

[27] Dutta S., Banerjee S. (2014). Exposure to Indoor Air Pollution & AMP.; Women Health. Environ. Urban. Asia.

[28] Maji K.J., Sarkar C. (2020). Spatio-Temporal Variations and Trends of Major Air Pollutants in China during 2015–2018. Environ. Sci. Pollut. Res. Int..

[29] Guo H., Gu X., Ma G., Shi S., Wang W., Zuo X., Zhang X. (2019). Spatial and Temporal Variations of Air Quality and Six Air Pollutants in China during 2015–2017. Sci. Rep..

[30] Ren L., Yang W., Bai Z. (2017). Characteristics of Major Air Pollutants in China. Ambient. Air Pollut. Health Impact China.

[31] Wang F., Wu M. (2021). Does Air Pollution Affect the Accumulation of Technological Innovative Human Capital? Empirical Evidence from China and India. J. Clean. Prod..

[32] Liu Z., Yu L. (2020). Stay or leave? The Role of Air Pollution in Urban Migration Choices. Ecol. Econ..

[33] Jia K., Chen S. (2021). Escaping from Pollution: Air Pollution and the Settlement Intentions of Floating Migrants in Chinese Cities. Migr. Stud..

[34] Li X., Chen H., Li Y. (2020). The Effect of Air Pollution on Children’s Migration with Parents: Evidence from China. Environ. Sci. Pollut. Res..

